# Anomalous Origin of the Right Coronary Artery From the Left Coronary Cusp Presenting as a Non-ST-Elevation Myocardial Infarction (NSTEMI)

**DOI:** 10.7759/cureus.30839

**Published:** 2022-10-29

**Authors:** Mohammad J Malik, Lucas Seibolt

**Affiliations:** 1 Cardiology, Philadelphia College of Osteopathic Medicine South Georgia, Valdosta, USA; 2 Interventional Cardiology, South Georgia Medical Center, Valdosta, USA

**Keywords:** coronary artery disease (cad), coronary artery atherosclerosis, adult congenital heart disease, interventional and structural cardiology, anomalous coronary arteries, non-st segment elevation myocardial infarction (nstemi)

## Abstract

Coronary artery anomalies are relatively uncommon in the general population with a roughly 1% incidence. Though oftentimes asymptomatic, these anomalies can be detrimental, resulting in myocardial infarction and even sudden cardiac death. Here, we present a case in which a 65-year-old male presented to the emergency room with substernal chest pain that radiated into his left arm. The patient’s cardiac enzyme panel revealed a troponin of 10.14 ng/mL and a creatine kinase-myocardial band (CK-MB) of 78.8 ng/mL. Furthermore, the patient’s electrocardiogram demonstrated normal sinus rhythm with no significant changes. Upon cardiac catheterization, his right coronary artery was found to originate from the left coronary cusp. Moreover, the anomalous artery demonstrated significant stenosis in its middle portion, which was presumably causing his elevated cardiac enzymes and anginal chest pain. The patient underwent successful percutaneous coronary intervention and was discharged the following day.

## Introduction

Coronary artery anomalies (CAAs) are characterized by the abnormal development of the three major coronary arteries. These include the right coronary artery, which normally arises from the right coronary sinus, and the left anterior descending and the left circumflex arteries, which arise from the left main coronary artery, which originates from the left coronary cusp [1]. The incidence of anomalous coronary arteries is thought to be about 1% and roughly 80% are considered benign [2]. The remaining 20% can be potentially life-threatening and are the second leading cause of sudden cardiac death (SCD) in young athletes [3]. CAAs can be further characterized by their origin and course. One of these variations includes the anomalous aortic origin of a coronary artery (AAOCA) [4]. This anomaly occurs when a coronary artery arising from the aorta has an abnormal origin or course. For example, a left coronary artery arising from the right coronary cusp or a right coronary artery arising from the left coronary cusp. AAOCA, in particular, has been associated with an increased risk of SCD [4]. Furthermore, AAOCA can follow various courses including intraarterial, subpulmonic, prepulmonic, retroaortic, or retrocardiac [5]. Here, we present a case in which a patient presented with a right coronary artery originating from the left coronary cusp and followed a retroaortic course.

## Case presentation

**Figure 1:**
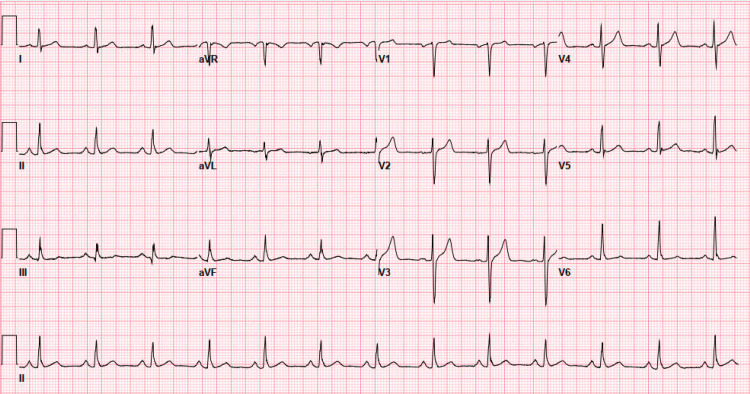
EKG demonstrating normal sinus rhythm along with the absence of any significant changes.

**Figure 2:**
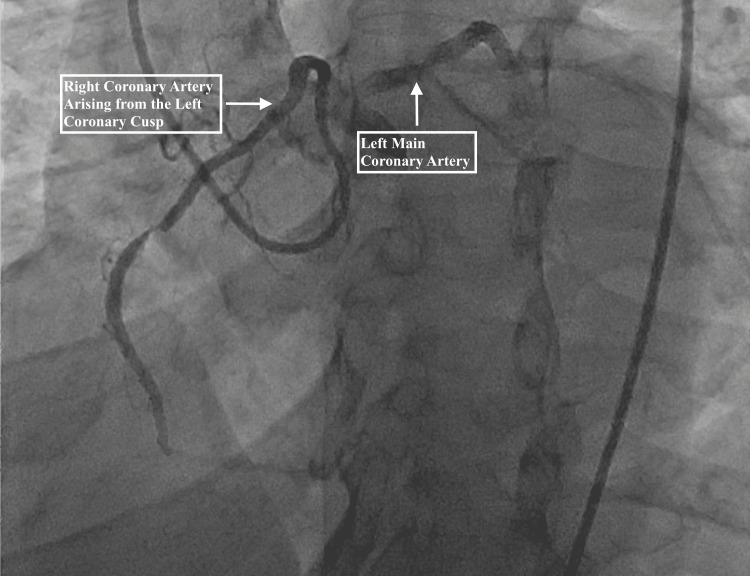
Coronary angiogram prior to drug-eluting stent placement reveals the patient's right coronary artery originating from the left coronary cusp.

**Figure 3:**
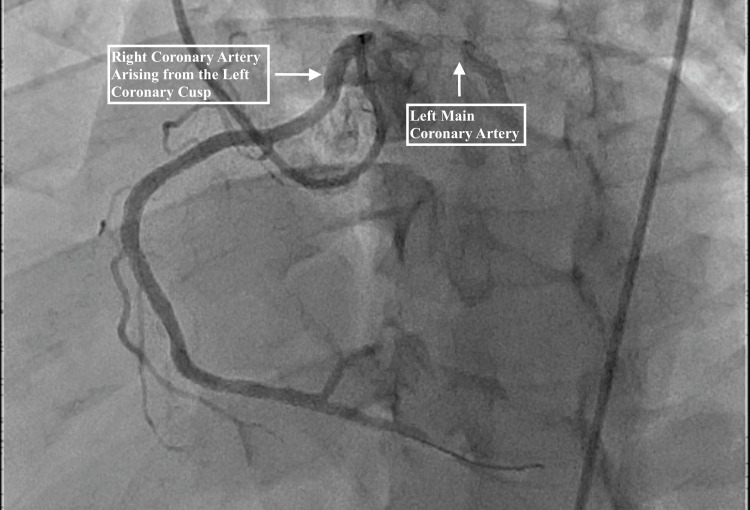
Coronary angiogram after successful placement of drug-eluting coronary artery stent.

A 65-year-old male with a past medical history of hypertension presented to the emergency room with substernal chest pain with radiation to his left arm. The patient’s chest pain was associated with nausea; however, he denied having shortness of breath or feeling diaphoretic. On admission, his cardiac enzymes were significantly elevated with a troponin of 10.14 ng/mL and a creatine kinase-myocardial band (CK-MB) of 78.8 ng/mL. An electrocardiogram (EKG) was also obtained, which revealed normal sinus rhythm with no significant changes (Figure 1). Thus, with the patient’s significantly elevated cardiac enzymes and normal EKG, he met the non-ST-elevation myocardial infarction (NSTEMI) criteria. The following day, the patient underwent cardiac catheterization at which point his anomalous right coronary artery was discovered (Figure 2). His anomalous right coronary artery demonstrated significant stenosis in its middle portion. The successful percutaneous coronary intervention was performed with the placement of an intraluminal drug-eluting stent. The patient was discharged the following day (Figure 3).

## Discussion

This case demonstrates an exceedingly rare presentation of coronary anatomy. It has been well-documented that an anomalous origin of coronary arteries can cause syncope, angina, and SCD [6]. Other causes of SCD include severe aortic valve stenosis [7]. The risk of SCD is further elevated if the anomalous coronary artery follows an intraarterial course, which is characterized by the artery coursing between the ascending aorta and the pulmonary artery trunk. Though the patient in this case had atherosclerosis of his coronary arteries, AAOCA has been known to cause the aforementioned symptoms in the absence of coronary artery disease, further exemplifying the clinical significance of such findings [8]. Moreover, studies have shown that anomalous coronary arteries may demonstrate accelerated atherosclerosis resulting in coronary artery disease [9,10]. The American College of Cardiology and the American Heart Association recommend repairing a symptomatic AAOCA. However, the management of asymptomatic patients with an AAOCA is not as clearly defined. Currently, there is a class IIb recommendation for repairing an asymptomatic anomalous right or left coronary artery from the contralateral coronary sinus that follows an intraarterial course [11,12]. Surgical correction of a left coronary artery from the right sinus is more strongly considered due to an increased risk of SCD [13]. Repairing such an anomaly involves reimplanting the right coronary artery directly into its appropriate sinus [11,12].

## Conclusions

Our patient presents a unique case of a right coronary artery arising from the left coronary cusp. His coronary angiogram revealed stenosis of his right coronary artery. While it is difficult to conclude that his right coronary artery occlusion was a direct result of its anomalous origin, studies have demonstrated anomalous coronary arteries having accelerated atherosclerosis. Though the management of patients with anomalous coronaries remains controversial, early identification and close monitoring of these patients is critical.
